# Investigation of validity evidence for the Coach Precompetitive Communication Questionnaire-Preference for measurement of collegiate athlete preferences of coach speech content and delivery

**DOI:** 10.3389/fspor.2025.1615784

**Published:** 2025-09-12

**Authors:** Corinne T. Zimmerman, Nicholas D. Myers, Robin S. Vealey

**Affiliations:** ^1^ Department of Kinesiology, Michigan State University, East Lansing, MI, United States; ^2^ Department of Sport Leadership and Management, Miami University, Oxford, OH, United States

**Keywords:** measurement, competition readiness, communication, coaching, athlete performance

## Abstract

**Introduction:**

Previous measures of athlete perception of pregame speech have centered around a two-dimensional structure of speech content (i.e., tactical and emotional), although psychometric evidence is limited. The Coach Precompetitive Communication Questionnaire – Preference (CPCQ-P) was developed to extend the two-dimensional model of pregame speech content by (a) including speech delivery and (b) allowing a general pregame speech factor. The purpose of this study was to investigate initial validity evidence for responses to the CPCQ-P under an exploratory bifactor approach at the athlete level.

**Methods:**

Participants were athletes (*N* = 264) at level-1 nested within NCAA varsity level teams (*G* = 36) at level-2. Participant survey responses were analyzed using an exploratory bifactor analysis with a general factor (i.e., pregame speech) and three grouping factors (i.e., tactical content, emotional content, delivery).

**Results:**

A four-factor model with three grouping factors and a general factor exhibited approximate to close fit to the data. Review of factor loadings provided initial evidence of validity for the internal structure of responses to the CPCQ-P.

**Discussion:**

This study expands the existing psychometric understanding of pregame speech within the collegiate sport context.

## Introduction

Researchers have conceptualized pregame speech as a coach-delivered address directed towards a team prior to competition (1). The content of pregame speech can be tactical and/or emotional in nature (2) and can be accompanied by nonverbal messaging (3). A survey developed by Vargas-Tonsing and Guan (2) investigated the athlete preferences of two types of verbal communication (tactical or emotional) given various competitive contexts. This survey did not include nonverbal aspects of pregame speech, nor was it a direct measure of pregame speech content. The Coach Precompetitive Communication Questionnaire-Preference (CPCQ-P) was created, as described by the current study, to expand the previous measurement of pregame speech by including measures for content (i.e., tactical or emotional) and delivery. Specifically, the CPCQ-P measures athlete preference for specific pregame speech behaviors displayed by their head coach on a general basis. Consistent with the current standards for educational and psychological testing (4), authors of this study sought to provide the necessary evidence of validity for responses to the newly developed survey. The focus of this study was centered on content-oriented and internal structure related evidence of validity.

The goal of pregame speech is to provide “last minute” thoughts that can benefit athletes prior to performance, such as increasing feelings of self-efficacy (i.e., an individual's’ belief in their ability to accomplish a task) (5). This understanding of pregame speech is rooted in Bandura's (6) four sources of self-efficacy: past experience, vicarious experience, verbal persuasion, and physiological or emotional sensation. Coaches use verbal persuasion to prepare their athletes for competition; verbal persuasion is a convenient and effective tool coaches can use to build athlete feelings of self-efficacy and collective efficacy (i.e., an individual's belief in the team's ability to accomplish a task) (7–9). In a team context, verbal persuasion allows coaches to provide their athletes with specific feedback from past performance that can be used for the upcoming competition. Additionally, verbal persuasion can be utilized to influence the affective states of athletes prior to competition. Research has shown that positive affect has been related to positive performance (10) and that athletes are particularly receptive to the expression of emotion from their coaches (3).

The specific verbal messaging utilized for verbal persuasion in the precompetitive context is considered the pregame speech content. Speech content can be tactical (i.e., in reference to specific skills or strategies) or emotional (i.e., in reference to specific emotions or motivations that will help the athletes perform). Speech delivery is considered the nonverbal (i.e., body language) or paraverbal (i.e., speech tone or expression) information that accompanies the content of pregame speech (11). For example, prior to a competition a coach might say to their team: “We have got to play tough.” The verbal content encourages athletes to strategically play strong and to emotionally remain grounded. The delivery behavior of this message might impact the athletes’ interpretation of that message. Thus, genuine enthusiasm can be taken for a positive display of emotion, while sharp yelling and the throwing of objects can be interpreted as negative.

Pregame speech has varying purposes for coaches and athletes. Coaches can use pregame speech as a means to organize their thoughts during their a pregame routine (1). Pregame speeches also allow coaches the opportunity to share their expectations for competitive strategy and effort with athletes and staff. For athletes, pregame speech can be used to increase competition readiness (3, 5, 11). Competition readiness includes but is not limited to, self-efficacy, collective efficacy (5, 8), motivation (12), focus, emotion and energy regulation (3, 13), and eliminating role ambiguity (14).

Pregame speech is central to promoting athlete competition readiness. Tactical speech content has been associated with decreasing role ambiguity by providing information that can direct athletes’ skillful performance (2, 15). Tactical content can be useful for facilitating coordination and task cohesion within collective team sports (e.g., soccer, basketball, hockey) where athletes need to work together to succeed (16, 17). Positive emotional content has been connected to motivation (12), energy, and emotional regulation (3, 5, 13).

Individual athlete preferences regarding pregame speech content can vary upon the competitive context (2, 18, 19). Researchers have found that elite youth athletes reported a desire for emotional content in pregame speech prior to playing a fierce rival, in contrast to a desire for more informational content when playing an unknown opponent (20). The consideration of athlete preferences before the delivery of a pregame speech may allow coaches to promote competition readiness more effectively.

However, athlete competition readiness is not achieved through verbal content alone. Researchers (3) found that nonverbal demonstrations of coach emotions can assist in meeting the emotional needs of higher recreational baseball and softball athletes prior to competition. The emotional expression of coaches can elicit affective, cognitive, and behavioral performance responses from athletes. The expression of positive emotions from coaches prior to competition has been associated with athlete expressions of happiness and successful team performance during the first half of competition.

A study of female hockey players indicated that these athletes value genuine displays of emotion by their coach during the pregame speech and will evaluate this type of speech more favorably (11). Bunning and Thompson (12) explored the coach behaviors that influence athlete motivation. Their inquiry found that the combination of coach communication and its associated nonverbal behaviors (i.e., tones, points of emphasis, body language) were valued enhancers of athlete motivation. These findings support the argument that the conceptual understanding of pregame speech should include the distinction between the verbal content itself and the delivery of that content. As such, the authors of this paper recommend that the study of pregame speech should include both content (i.e., tactical and emotional) and delivery.

The construct of pregame speech has been studied in a variety of ways. Qualitative inquiry from Bloom (1) provided insight regarding coach perspectives on the use of pregame speech. Themes identified included coaches keeping an even temper, staying focused, and only highlighting a few key points from practice that related to the competition. Delivery and content—both emotional and tactical—were described by coaches as valuable components of their pregame speeches. The use of open-ended questions to investigate preferred aspects of pregame speech further supported the distinction of tactical and emotional speech content made by athletes (11, 12, 20).

Vargas-Tonsing and Guan (2) explored the quantitative measurement of the athlete experience of pregame speech via the Speech Content Preference Measure. This survey was used to understand the amount of informational and emotional content that athletes preferred in their coaches’ pregame speeches given specific competitive contexts. The survey was comprised of two sections: informational content and emotional content. Each section presented participants with nine competitive scenarios. Participants were asked “How much information or emotional content would you want to hear in your coach's pre-game speech when..” and were directed to respond on a five-point Likert scale (1 = very little to 5 = very much). Results provided summative comparisons of the data collected from collegiate athletes.

However, the Speech Content Preference Measure did not directly measure the unique construct of pregame speech. This survey was designed to specifically measure the athlete preference of the two suggested types of verbal content. While a principal component analysis provided evidence that supported a two-component distinction of speech content, this study did not provide an additional factor analysis that would suggest the ability to directly measure these unique components. While the Speech Content Preference Measure is a useful tool to gather data about athlete preferences given specific competitive situations, its psychometric limitations and the absence of nonverbal communication ultimately constrict the understanding of pregame speech. The direct measure of speech content and inclusion of pregame speech delivery would allow for a more holistic conceptualization of pregame speech as its own unique factor.

The Coach Precompetitive Communication Questionnaire- Preference (CPCQ-P) was developed by authors to measure the general preferences of athletes for pregame speeches by their current head coach. While, the Speech Content Preference Measure provided athlete self-reports of preferred pregame speech content for a given context, the CPCQ-P expands upon this previous survey with the addition of speech delivery and measurement of the specific pregame speech behaviors. The CPCQ-P asks athletes to indicate how often they would prefer their head coach to utilize specific pregame speech behaviors. The concept of preference is understood as a judgement of comparison or ranked opinions for specific behaviors (21). The CPCQ-P uses this understanding to operationalize athlete preference of coach behaviors (i.e., the individual opinion regarding specific pregame speech behavior).

The inclusion of delivery within the operational understanding of pregame speech provides rationale to expand the measurement of pregame speech to reflect this new understanding. Additionally, the assumption that pregame speech is used as a tool to influence athletes prior to competition suggests that the ability to have knowledge of athlete pregame speech preferences is valuable for researchers, coaches, and practitioners. The CPCQ-P is a survey used to understand athlete perception of pregame speech and to identify what components are found valuable to their performance preparation. The purpose of this study was to investigate initial validity evidence for responses to the CPCQ-P under an exploratory bifactor approach at the athlete level.

## Method

Permission from an institutional review board was acquired prior to the start of this study. The desired population for this study was NCAA varsity level athletes from interactive team sport (e.g., basketball, volleyball, etc.) due to the collaborative nature of these teams prior to competition. To gain access to this population researchers sent an invitation to head coaches of interactive sport teams with email addresses publicly listed on their athletic department's website. The invitation email asked the head coach if they would be interested in having their athletes participate in a research study focused on pregame speech, explained what would be asked of their athletes, and offered two data collection modalities (i.e., in-person or online). Coaches were contacted three times before they were removed from the email list due to non-response. Data collection occurred in-person or online depending on the preference of the head coach of participating teams. In-person data collection procedures involved on-campus visits following a team practice or training session. Researchers administered paper surveys to participants in the training environment. Researchers introduced themselves, the research topic, and explained the informed consent materials to athletes during in person data collection procedures. Online participation involved researchers sending email invitations directly to participating athletes upon receiving an email list from the head coach. The invitation included a description of the study, a reminder that participation was voluntary, assurance that all responses were confidential, and the link to an online survey tool (Qualtrics). Athletes were encouraged to complete the survey within a week of receiving the invitation. Communication with athletes ceased following two reminder emails sent during a two week window following initial contact. Surveys used for data collection were identical regardless of data collection modality. Completion of the survey took no more than 10 min.

Athletes needed to be at least 18 years of age and actively participating in their varsity level interactive team sport to participate in the current study. Participants were 264 NCAA varsity level athletes from the Midwestern United States. Participants (149 female, 137 male) ranged from 18 to 23 years of age (*M* = 19.64, *SD* = 1.33). The current sample of athletes had an average of 12.4 years of total sport experience and an average 1.69 years with the head coach of their current team. The majority of participants were within their first three years of college (80.3%). A majority of participants identified as white/Caucasian (*n* = 244), with the remainder identifying as Black/African American (*n* = 12), Asian (*n* = 4), multiracial (*n* = 9), Hispanic (*n* = 9), or Native/Indigenous American (*n* = 4). Participants represented 36 interactive sport teams that included sports such as soccer, basketball, volleyball, baseball, lacrosse, softball, and hockey.

### Coach Precompetitive Communication Questionnaire-Preference

Authors of this paper developed a 12-item survey that measures athlete preferences of pregame speech (see Table 1). Content development was inspired by the survey instructions of the Speech Content Preference Measure and further rooted in the subsequent literature regarding pregame speech. Content experts from the fields of sport and performance psychology and coaching research were consulted in the development process. Experts were asked to review survey items and asked to organize them based on pregame speech factors (i.e., tactical content, emotional content, or delivery). With the consideration of expert insight and subsequent literature, pregame speech was operationalized as coach-delivered verbal persuasion that utilizes tactical content, emotional content, and nonverbal delivery to share a specific message prior to athletic competition.

**Table 1:** Percentage of observed responses to each coach Precompetitive Communication Questionnaire-Preference survey item by item response.

CPCQ-P survey dimensions	Item of number and content	Level of preference				
		Never	Rarely	Sometimes	Often	Always
Tactical content	Prefer_1: Talk about our team's specific strategy	.7%	3.5%	11.9%	32.5%	43.7%
	Prefer_2: Talk about specific skills and/or techniques	3.5%	9.4%	22.0%	29.7%	27.6%
	Prefer_3: Talk about our opponent (likely strategies or skills)	4.2%	9.1%	18.9%	26.6%	33.6%
	Prefer_ 6: Talk about the importance of the competition	2.8%	10.8%	24.8%	18.5%	35.3%
Emotional content	Prefer_4: Talk about their confidence in us	0.00%	2.4%	3.8%	21.3%	64.7%
	Prefer_5: Talk about our preparation and readiness	.3%	3.1%	12.2%	30.4%	46.2%
	Prefer_7: Try to energize the team	1.0%	2.8%	13.6%	22.4%	32.4%
	Prefer_8: Try to relax the team	2.1%	7.7%	22.0%	29.0%	31.5%
Delivery	Prefer_9: Is confident when talking to us	.3%	1.4%	2.4%	17.5%	70.6%
	Prefer_10: Is calm when talking to us	1.7%	3.8%	17.8%	22.0%	45.8%
	Prefer_11: Is emotional when talking to us	13.6%	19.2%	28.7%	14.3%	16.4%
	Prefer_12: Is genuine and real when talking to us	.3%	0%	4.5%	14.7%	72.7%

There are three specific factors proposed and defined in the CPCQ-P. Tactical Content (items 1–3, and 6), is understood as the verbal description of specific skills or strategies relevant to the competition. This four-item subscale asked athletes to consider their coaches’ descriptions of team specific strategy, specific skills or techniques, the opponent's likely skills or strategies, and the importance of the competition. Emotional Content (items 4–5 and 7–8) is considered the specific feelings or motivations verbally discussed by a coach in their pregame speech. The four items of the CPCQ-P that measure Emotional Content items included athletes’ preference of their coach talking about their confidence in the team, the team's preparation, and using words to energize or relax the team. Lastly, Delivery (items 9–12) is the nonverbal messaging that accompanies the verbal content shared by the coach. These items included coaches’ display of confidence, calmness, emotion, and authenticity during their pregame speech.

The survey instructions asked participants to indicate “how often you would prefer your head coach to say or do…” followed by a specific item in their precompetitive talks. For example, an item for the Tactical Content subscale would read “Talk about our team's specific strategy.” Participants responded on a five-point Likert scale regarding how often they would prefer their current head coach to use specific pregame speech behaviors (1 = prefer that coach never does this to 5 = always prefer that coach does this).

### Data analysis

Statistical models were fit in M*plus*, version 8.11 (22). Weighted least squares mean- and variance- adjusted (WLSMV) estimation for categorical variable methodology (23) used as is consistent with recent recommendations in kinesiology (24). Missing data were treated as missing at random under a full maximum likelihood approach (25) consistent with the recommendations for application in kinesiology (26).

**Figure 1:**
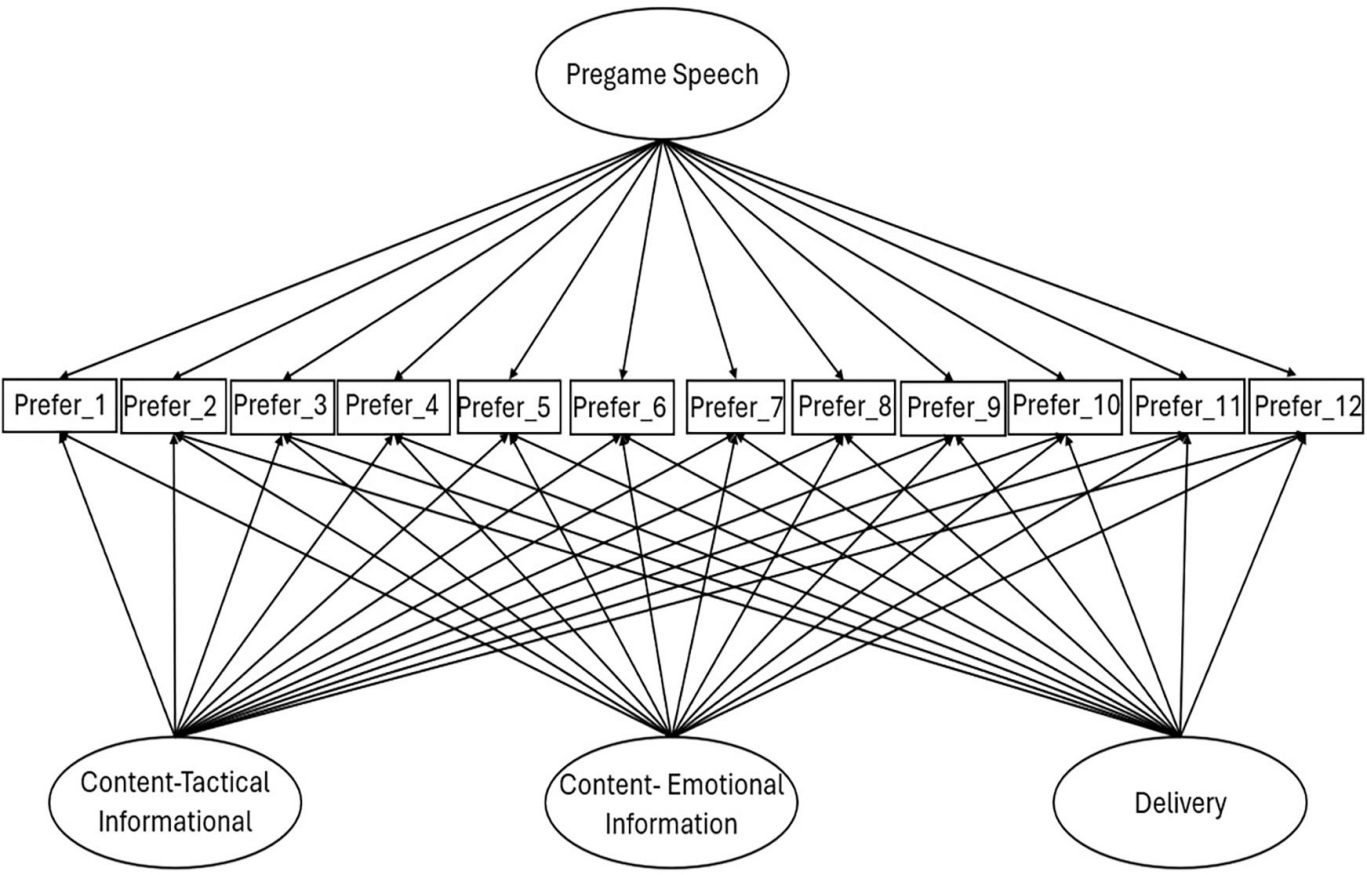
Conceptual image of the exploratory bifactor model of pregame speech.

An exploratory bi-factor model (EBFA; 27) was selected for data analysis consistent with the recent recommendations for applications in kinesiology (27). Specifically, Myers and colleagues suggest that an EBFA may be beneficial if there is theoretical rational for a general factor and specific grouping factors in situations when a more restrictive analysis (e.g., correlated first-order analyses with fewer dimensions) would not be practical. In this study, the lack of complete *a priori* data prevented a confirmatory bi-factor model to be utilized (28). An exploratory bi-factor approach allowed for all three group factors—tactical content, emotional content, and delivery—to directly influence all twelve survey items. All twelve items were simultaneously influenced by the general variable of pregame speech (see Figure 1). Loading matrices for both group and general factors were allowed to naturally emerge (27, 28).

Orthogonal target rotation (29) was used in the EBFA. The use of orthogonal rotation was consistent with historical underpinnings of the bi-factor model (30). The target matrix was fully specified (e.g., each tactical content item was targeted to have a factor loading = .75 on tactical content and = .00 on all other group factors) based on recommendations for using target rotation in practice (31, 32). The targeted values were derived from an iterative approach guided by human judgment (33).

### Effect size

Effect size was considered via two recommendations. The first approach was through the calculation of the percentage of common variance explained [PCVE; (34)]. This value is derived from the ratio sum of squares of the standard factor loading of the given factor and the sum of squares of the standard factor loadings for general and grouping factors. The second approach was through factor loadings that demonstrated statistical significance and had an absolute value greater than or equal to .20. The recommended value is somewhat arbitrary, but is consistent with Jennrich and Bentler (35).

### Model-data fit

An initial exploratory factor analysis (EFA) was generated to explore the factor structure of initial responses to the CPCQ-P. Indices of model data fit considered for the EFA and final EBFA models were: *χ*^2^, RMSEA (Root Mean Square Error of Approximation), SRMR (Standardized Root Mean Square Residual), CFI (comparative fit index), and TLI (Tucker Lewis Index). The chi-square exact fit test suggests that *p* > .05 is indicative of good model-data fit. RMSEA is a fit index of approximate model that adjusts for model complexity. A test value of less than .05 suggests close model-data fit. An SRMR value less than .08 also indicates good model-data fit. The CFI and TLI are both incremental fit indices, with the TLI adjusting for model parsimony. For both these indices a value greater than .95 represents close model-data fit. These values reflect the general recommendations for model-data fit (36). To ensure the retention of a statistically significant model for EBFA, a formal comparison of the change in chi-square exact fit was conducted for nested data and is described in the following section.

## Results

Table 2 provides polychoric correlations of the 12 CPCQ-P items. No differences relative to data collection format was observed within the data. An EFA model was generated to explore the factor structure of initial responses to the CPCQ-P. Models with an increasing number of factors (*m* = 1, 2, etc.) were fit to the data. Note that an EBFA is equivalent to an EFA with a bi-factor rotation method, when *m* ≥ 2 (28). A formal comparison between nested models found statistically significant differences between simpler and complex models. These results supported the retention of the more complex model and indicated that a four-factor model possessed the better fit to the data (see Table 3). There was evidence for close to adequate fit of the four-factor EBFA: *χ*^2^ (24, *N* = 264), 42.321, *p* = .012, RMSEA = .054 (CI_90%_ = .025-.080), *p* = .378, SRMR = .004, CFI = .975, and TLI = .931.

**Table 2:** Polychoric correlations for all study variables.

Variables	1	2	3	4	5	6	7	8	9	10	11	12
1. prefer_1	–	–	–	–	–	–	–	–	–	–	–	–
2. prefer_2	.60	–	–	–	–	–	–	–	–	–	–	–
3. prefer_3	.58	.47	–	–	–	–	–	–	–	–	–	–
4. prefer_4	.07	.04	.05	–	–	–	–	–	–	–	–	–
5. prefer_5	.21	.18	.04	.61	–	–	–	–	–	–	–	–
6. prefer_6	.09	.17	.25	.30	.49	–	–	–	–	–	–	–
7. prefer_7	.13	.16	.14	.46	.27	.40	–	–	–	–	–	–
8. prefer_8	.18	.24	.07	.37	.26	.24	.55	–	–	–	–	–
9. prefer_9	.22	.23	.15	.59	.47	.22	.42	.44	–	–	–	–
10. prefer_10	.05	.25	-.07	.29	.26	.15	.07	.62	.47	–	–	–
11. prefer_11	.17	.24	.34	.05	.10	.35	.29	.30	.06	.21	–	–
12. prefer_12	.26	.27	.17	.49	.30	.05	.34	.32	.66	.33	.12	–
M	4.25	3.74	3.83	4.61	4.29	3.79	4.33	3.87	4.70	4.16	3.01	4.73
SD	.87	1.11	1.16	.70	.85	1.16	.92	1.05	.64	1.01	1.29	.59
Range	1–5	1–5	1–5	2–5	1–5	1–5	1–5	1–5	1–5	1–5	1–5	1–5

**Table 3:** Number of factors warranted to explain responses to the coach Precompetitive Communication Questionnaire-Preference (CPCQ-P).

	Goodness of fit					Nested model comparison	
Model	*χ*^2^ (df)	RMSEA [CI90%]	CFI	TLI	SRMR	Model compared	Δχ^2^(Δdf)
Model 1: m = 1	293.908 (54)^***^	.130 [.115–.144]	.673	.601	.142	–	–
Model 2: m = 2	153.973 (43)^***^	.099 [.082–.116]	.849	.768	.097	Model 1 vs. model 2	156.514 (11)^***^
Model 3: m = 3	97. 914 (33)^***^	.086 [.067–.106]	.912	.823	.075	Model 2 vs. model 3	74.491 (10)^***^
Model 4: m = 4	42.321 (24)*	.054 [.025–.08]	.975	.931	.044	Model 3 vs. model 4	63.572 (9)^***^

* *p* < .05, **p < .01, ***p < .001.

A *post-hoc* power analysis for model-data fit (37, 38) was conducted as advocated in exercise science (39, 40) using an online utility (41). Alpha was set to .05. Degrees of freedom

(df) were set to 24 consistent with the accepted model. Sample size was set to 264. Population model-data fit (*ε*) was set to .10 in the null condition (*ε*_0_) to represent a boundary for poor fit consistent with general methodological recommendations (42) and a level of misfit that likely would be judged as problematic during instrument development. Population model-data fit was set to .05 in the alternative condition to represent close fit consistent with general methodological recommendations. Power estimation equaled .96.

### General factor: pregame speech behavior

The four-factor EBFA allowed for all 12 items of the CPCQ-P to load onto the general factor of Pregame Speech Behavior and each of the three group factors (see Table 4). Standardized factor loadings ranged from −0.26 to 0.56. Only five of the 12 items of the CPCQ-P demonstrated a significant loading on the general factor. Three of those items were intended to measure Tactical Content and included factor loadings that ranged from .22 to .43. Two items intended to measure Delivery also possessed meaningful factor loadings from Pregame Speech and ranged from .51 to .57. The overall Pregame Speech Behavior factor accounted for 18% of the common variance demonstrated in the 12 items.

**Table 4:** CPCQ-P under EBFA with orthogonal target rotation.

	General factor				Group factors				
	Pregame speech		Tactical content		Emotional content		Delivery		
Item/PVCE	*λ*	*SE*	*λ*	*SE*	*λ*	*SE*	*λ*	*SE*	*R* ^2^
Prefer_1	.43	.11	.70	.06	–	–	–	–	.72
Prefer_2	.25	.12	.59	.05	–	–	.33	.07	.53
Prefer_3	.22	.08	.69	.07	–	–	–	–	.53
Prefer_4	–	–	–	–	.73	.16	–	–	.73
Prefer_5	–	–	–	–	.62	.07	–	–	.49
Prefer_6	–	–	.51	.10	.74	.09	–	–	.88
Prefer_7	–	–	–	–	.45	.04	.21	.08	.30
Prefer_8	–	–	–	–	.32	.10	.70	.06	.61
Prefer_9	.51	.18	–	–	.48	.15	.45	.07	.69
Prefer_10	–	–	–	–	–	–	.78	.10	.68
Prefer_11	–	–	.41	.08	–	–	.24	.08	.29
Prefer_12	.57	.13	–	–	–	–	.39	.05	.55
PVCE		18%				82%			

*λ*, pattern coefficient; PCVE, percentage of common variance explained. Estimated factor loadings that were not statistically significant (*p* > .05) and | *λ* | <.20 were omitted from the table.

### Group factors

The analysis of group factors involved the free estimation of responses to the 12 survey items on the grouping factors of Tactical Content, Emotional Content, and Delivery. The combination of grouping factors accounted for 82% of the common variance explained. The following results report both the factor loadings of responses to anticipated items and any additional cross-loadings of responses to additional items.

#### Tactical content

The Tactical Content subscale was comprised of four items (Prefer_1, Prefer_2, Prefer_3, and Prefer_6) from the CPCQ-P. The standardized factor loadings from the first group factor, Tactical Content, on the targeted items ranged from .51 to .70. An additional item (intended to load on Delivery) demonstrated a significant factor loading from Tactical Content (*λ* = .41). These results suggest that higher responses on the Tactical Content subscale would indicate higher preference for Tactical Content in pregame speeches.

#### Emotional content

The Emotional Content subscale was made up of 4 items (Prefer_4, Prefer_5, Prefer_7, and Prefer_8). The standardized factor loadings from the second group factor, Emotional Content, on the targeted items ranged from .32 to .74. Two additional items (item 6 and item 9) had meaningful factor loadings that ranged from .48 to .74. Overall, positive factor loadings onto Emotional Content would suggest athletes’ increased preference for Emotional Content in pregame speech.

#### Delivery

The last grouping factor, Delivery, was comprised of 4 items (Prefer_9–12). The standardized factor loadings from this group factor on the targeted items to measure Delivery ranged from .24 to .78. There were an three additional items that had meaningful cross-loadings on to this grouping variable (items 2, 7, and 8) that ranged from .21 to .70.

## Discussion

The purpose of this study was to investigate initial validity evidence for responses to the CPCQ-P under an exploratory bifactor approach at the athlete level. The CPCQ-P expands on the two-factor approach of pregame speech previously used by Vargas-Tonsing and Guan (2) by including measurement items of speech delivery behaviors. Overall, the significant loadings of corresponding item responses indicated initial evidence of validity for the internal structure of the responses to the CPCQ-P and its three subscales. Indices of model-data fit showed support for the bifactor model of pregame speech that includes a general factor (pregame speech) and three group factors (tactical content, emotional content, and delivery).

This study investigated the evidence for validity of the internal structure of the responses to the CPCQ-P. This specific type of evidence of validity has been described as “…the degree to which relations among test items and test components conform to the [test] construct” (4). An EFA and formal comparison of nested models determined that a four-factor model structure demonstrated better data fit, providing support for the utilization of the EBFA for further analysis. Myers and colleagues (27) provided a strong case for the utility of the bifactor model in sport, exercise, and performance psychology, especially when a theoretical case for a general factor can be made. In the context of this study, pregame speech can be understood as an address given by a head coach to their athletes prior to competition (1, 7, 13). Additionally, the components of pregame speech can have their own unique effect on athlete interpretation as demonstrated by the research of speech content (2, 19) and delivery (3, 10, 11). It is logical that pregame speech exists as both a general factor as well as various specific factors. The findings of the EBFA support the use of the CPCQ-P as a direct measure of the general factor of pregame speech, rather than indirectly through related construct subscales.

Athlete responses to the CPCQ-P were indicative of preferences for a general pregame speech factor as well as the proposed grouping factors (i.e., tactical content, emotional content, and delivery). Previous quantitative research of pregame speech has isolated athlete perceptions of tactical and emotional content (2, 20) and delivery (3). While the isolated study of group factors can provide meaningful information for practical application, these factors are rarely used independent of one another. Coach communication is complex and multifaceted construct that relies on the experience and interpretation of simultaneous verbal and nonverbal messaging (13, 16, 43). Coaches are simultaneously using pregame speech content and delivery to impact athlete competition readiness (5, 15). For example, a soccer coach could attempt to calm nervous athletes ahead of a match by using a soothing tone and reminding them of their fundamental skill proficiency. Together, the speech content and delivery convey the intended message of the coach and create the overall athlete experience of pregame speech. Therefore, the bifactor nature of pregame speech, as identified within the current study, confirms the complexities previously been identified in the coach communication literature (16, 43) and expands what has previously been demonstrated in the pregame speech literature (2).

Of the 12 items of the CPCQ-P, five items possessed significant factor loadings onto pregame speech. Three of those items were intended to load onto the tactical content group factor. These items asked athletes to consider how often they preferred their head coach to discuss specific team strategies, specific skills, or the specific strategy of their opponent. This is supported by the previous findings of Vargas-Tonsing and Guan (2), which indicated that athletes prefer tactical content in specific conditions. Items of the delivery subscale also demonstrated significant loadings onto the general factor (i.e., coach appearing confident or genuine in their address), which is consistent with recent research that has indicated that athletes prefer coach authenticity (11) and positive displays of emotion and confidence (3, 8, 10, 18, 19). The results of the current study showed that given the items of the CPCQ-P, collegiate athlete responses reflected generally higher scores of pregame speech preference when higher scores of tactical information and delivery were recorded. The current study also demonstrates that intercollegiate athletes’ experience of pregame speech is multifaceted and dependent on various verbal and nonverbal communication factors, providing evidence for the inclusion of delivery (i.e., nonverbal messaging) in the operational definition of pregame speech.

### Grouping factors

The CPCQ-P intended to measure pregame speech utilizing three previously identified subscales. The twelve items of the CPCQ-P were developed primarily through review of the current research (2, 3, 5, 7, 11, 18) and the input of content experts. All targeted items demonstrated significant loadings onto their intended grouping factors providing empirical evidence of the validity of responses to the survey.

Tactical content in the context of this survey was identified by the verbal articulation of team strategy, skills and techniques, an opponent's strategy, and statistical importance of the given competition. Research has shown that in various competitive contexts the inclusion of specific tactical information can be useful in preparing athletes for competition (2, 5, 17–20). For example, if a team is participating in a championship game or a rematch of a rival opponent, some athletes might prefer their coach to utilize more tactical content in their pregame speech (18, 20).

The CPCQ-P items for emotional content also indicated evidence for the internal validity of responses to that subscale. Emotional content for the CPCQ-P is understood as the verbal expression of a coach's confidence in their team, their belief in team preparation, and verbal attempts to energize and/or relax their team. This subscale is not an exhaustive list of emotional content, rather it is a distinct reflection of previous research (2, 18, 20). Significant cross loadings were found in coach nonverbal expression of confidence during speech delivery and their verbal articulation of the importance of the competition. It is likely that the distinction between the verbal and nonverbal expression of confidence is nuanced and not easily distinguishable among participants. Athletes may interpret their coach's verbal and nonverbal expressions of confidence similarly as the intended message is the same (3).

The final CPCQ-P subscale of delivery was intended to measure the nonverbal and paraverbal messaging used by head coaches during their pregame speeches. Items of this subscale referred to coach displays of confidence, calmness, general sense of emotion, and genuine authenticity in the delivery of pregame speech. All intended items loaded appropriately onto this factor. The item for displays of emotion demonstrated a weaker loading onto the factor of delivery in relation to the other items. This may be because participants may have differing interpretations of what “seeming emotional” is or the item itself is too vague. There is research on how the emotions of head coach can impact the emotional state of athletes prior to competition (3, 10, 11, 20). Specifically, athletes have reported that authentic displays of emotion from their head coach are likely to impact their perception of the effectiveness of the pregame speech, regardless of its content or the game context (11).

As previously mentioned, various items demonstrated significant cross-loadings onto the grouping factors. From a statistical perspective, significant cross-loadings are expected within the EBFA as the analysis allows for loadings to be freely estimated across all factors (44). Loadings that are freely estimated assist in the development of a better model of the data by providing information regarding the relationships between survey items and model factors (28). From a theoretical perspective, such cross-loadings are to be expected as speech content and speech delivery are perceived simultaneously and may be challenging to distinguish (3, 10). The information provided by study results will inform further item development of the CPCQ-P.

### Limitations and future directions

The authors of this study are aware of several limitations that may affect the interpretation of results. The context of this study was within intercollegiate, interactive sports teams. The selection of this sample was deliberate, as members of interactive sports teams prepare for competition together and are commonly addressed as a group by the head coach prior to the competition (1). However, future research should consider the nuance of the precompetitive address of coaches to collegiate athletes participating in coactive sports (i.e., gymnastics, track and field, etc.). The importance of precompetitive addresses in in these sporting contexts is lesser known and could be a valuable addition to the field of knowledge.

An additional limitation of this study involves the timing of data collection. Data were collected at various time points within participating teams’ competitive seasons due to team availability. Participants were asked to think about the typical coach pregame speech behaviors they have observed their current coach using. While this was the intent of the current study, it is recommended that the CPCQ-P be used at various time points throughout a season to investigate athlete preference throughout the season or in relation to a specific competitive context (2, 20). Additional variables of interest include the role of the pregame speech speaker within the team, the competitive level of the athletes, athlete and coach gender, coach purpose for pregame speech use (45), and the athlete perceptions of the coach-athlete relationship (11, 46).

Exploring additional covariates may provide a deeper understanding of the impact pregame speech can have on athlete competition readiness. For example, the coach-athlete relationship is a task-focused, bi-directional relationship between coaches and athletes intended to provide social support for goal attainment and relational development of all parties (47). The compatibility of the coach-athlete relationship has been associated with athlete perceptions of coach communication and athlete satisfaction (43, 47, 48). Therefore, it is logical that the coach-athlete relationship may influence athlete perceptions of coach pregame speeches and ultimately the effectiveness of pregame speeches on athlete precompetitive readiness. This theoretical construct and others, such as theories of motivation (43, 45, 49–51), are likely to be contributing factors that influence the use, experience, and effectiveness of pregame speech. Future research is needed to explore these possibly interconnected factors. Such research could provide relevant knowledge that may assist coaches in their ability to use pregame speech effectively to benefit athletes performance.

## Conclusions

The current study provided initial validity evidence for the responses of collegiate athlete preferences of general head coach pregame speech behaviors with the CPCQ-P. These findings help extend the empirical knowledge of pregame speech through the evidence of observed model data fit of an EBFA. This model implies that the general factor of pregame speech can be measured in addition to its grouping factors of tactical content, emotional content, and speech delivery. Further development of the CPCQ-P will provide numerous benefits to both research and applied settings within the context of collegiate athletics. Such a measurement will allow researchers and practitioners the ability to gain information regarding collegiate athlete preferences for the pregame speeches given by their coaches. While preferences are individualized, having a general understanding of what athletes prefer can aid coaches in their pre-competition preparation and allow them to deliver pregame speeches that can positively affect athlete competition readiness.

## Data Availability

The raw data supporting the conclusions of this article will be made available by the authors, without undue reservation.
